# Crystal Structure of Aura Virus Capsid Protease and Its Complex with Dioxane: New Insights into Capsid-Glycoprotein Molecular Contacts

**DOI:** 10.1371/journal.pone.0051288

**Published:** 2012-12-14

**Authors:** Megha Aggarwal, Satya Tapas, Preeti, Anjul Siwach, Pravindra Kumar, Richard J. Kuhn, Shailly Tomar

**Affiliations:** 1 Department of Biotechnology, Indian Institute of Technology, Roorkee, Roorkee, India; 2 Department of Biological Sciences and Bindley Bioscience Center, Purdue University, West Lafayette, Indiana, United States of America; University of Pennsylvania School of Veterinary Medicine, United States of America

## Abstract

The nucleocapsid core interaction with endodomains of glycoproteins plays a critical role in the alphavirus life cycle that is essential to virus budding. Recent cryo-electron microscopy (cryo-EM) studies provide structural insights into key interactions between capsid protein (CP) and trans-membrane glycoproteins E1 and E2. CP possesses a chymotrypsin-like fold with a hydrophobic pocket at the surface responsible for interaction with glycoproteins. In the present study, crystal structures of the protease domain of CP from Aura virus and its complex with dioxane were determined at 1.81 and 1.98 Å resolution respectively. Due to the absence of crystal structures, homology models of E1 and E2 from Aura virus were generated. The crystal structure of CP and structural models of E1 and E2 were fitted into the cryo-EM density map of Venezuelan equine encephalitis virus (VEEV) for detailed analysis of CP-glycoprotein interactions. Structural analysis revealed that the E2 endodomain consists of a helix-loop-helix motif where the loop region fits into the hydrophobic pocket of CP. Our studies suggest that Cys397, Cys418 and Tyr401 residues of E2 are involved in stabilizing the structure of E2 endodomain. Density map fitting analysis revealed that Pro405, a conserved E2 residue is present in the loop region of the E2 endodomain helix-loop-helix structure and makes intermolecular hydrophobic contacts with the capsid. In the Aura virus capsid protease (AVCP)-dioxane complex structure, dioxane occupies the hydrophobic pocket on CP and structurally mimics the hydrophobic pyrollidine ring of Pro405 in the loop region of E2.

## Introduction

Alphaviruses are members of *Togaviridae* family, possessing single-stranded positive-sense genomic RNA. They are causative agents of diseases ranging from mild fever to harsh encephalitis and may also be a menace of bioterrorism [1], [2]. In recent years, Chikungunya virus, an arthritogenic alphavirus, has been considered as an emerging threat to human health, which may lead to epidemic [3]–[5]. Hence, there is an urgent need for development of effective antiviral therapies and drugs against alphaviruses. Also, complete understanding of alphavirus budding will provide valuable information, as the budding process is a potential drug target.

Alphaviruses contain a nucleocapsid core surrounded by the lipid envelope through which spike glycoproteins penetrate. The nucleocapsid core is formed by the encapsidation of RNA by CP which consists of two domains: the amino-terminal domain that is involved in RNA binding and the carboxyl-terminal domain that possesses protease activity [6]–[8]. The E2 glycoprotein interacts with the nucleocapsid complex via a hydrophobic pocket present in the carboxyl-terminal region of the CP that leads to budding of alphaviruses [9]–[16]. Earlier investigations have shown that different regions in the cytoplasmic tail of E2 (cdE2) are involved in CP-glycoprotein interaction [17]–[19]. In this view, the conserved Y-X-L motif in E2 glycoprotein has been suggested to play a direct role in the interaction with the hydrophobic pocket of CP [20], [21]. Additionally, the crystal structure of CP from Sindbis virus was found to contain the solvent-derived dioxane in its hydrophobic pocket [22]. This suggested that dioxane or similar molecules may be able to enter the pocket and prevent capsid–E2 binding. In order to target and disrupt CP-glycoprotein interactions, dioxane based synthetic antiviral compounds have been designed against Sindbis virus based on the crystal structure of Sindbis virus CP containing dioxane in the hydrophobic pocket [23], [24]. In recent years, extensive studies on the CP-glycoprotein interaction in alphaviruses have riveted attention to further investigate and formulate new antiviral molecules [25], [26], [27]. In fact, in these studies, the pseudo-atomic model of Sindbis virus and E2 mutational studies revealed loop regions and other conserved residues that are essential to the interaction. Furthermore, three different contact regions in the capsid protein were identified, two of which consist of the exposed loops at the surface [26]. However, in order to evaluate the potency of CP-glycoprotein interaction, a more comprehensive study at the molecular level is essential.

Recently, the cryo-electron microscopic structure of Venezuelan equine encephalitis virus (VEEV) was determined at 4.4 Å resolution and reveals the arrangement of trans-membrane helices and cytoplasmic tails of E1 and E2 glycoproteins [27]. According to this cryo-EM structure, the linker region (residues 115–124) of CP was in the form of an α-helix and overlaps the region (residues 109–125) expected to interact with the 60S ribosomal subunit of host cell during disassembly of nucleocapsid. Interestingly, this linker region is not found to be in helical form in any known crystal structures of alphavirus CP. Moreover, it was previously assumed that the cytoplasmic domain of E1 has no role in CP-glycoprotein interactions [28]. However, recent findings provide evidence for the interaction between the cytoplasmic tail of E1 and CP, suggesting that E1 may also have a role in the viral budding process [26]. Furthermore, a charged interaction has also been reported to occur between the amino terminus of the cytoplasmic tail of E2 and CP [25]. Nonetheless, both the molecular interaction of E2 with the nucleocapsid core and the role of E1 glycoprotein in budding through interaction with CP are still not clearly understood. Additional structural investigations of CP-glycoprotein interactions from different alphavirus members are required for a detailed understanding of the budding process, which could be beneficial in the development and design of new drug molecules against alphavirus infection.

Here, we report the high-resolution crystal structures of the post-cleavage state of the AVCP and dioxane-bound AVCP at 1.81 Å and 1.98 Å resolution respectively. Homology models of Aura virus E2 and E1 were generated and validated. The mode of interaction of these glycoproteins with the capsid was investigated at the molecular level by comparing sequences and structures from different alphaviruses. The role of various conserved residues involved in CP-glycoprotein interaction and the basis for disruption of these interactions by binding of dioxane in the hydrophobic pocket of CP has been proposed.

## Materials and Methods

### Materials

For protein purification, Ni-NTA beads were purchased from Qiagen and imidazole (low absorbance at 280 nm) was obtained from Acros. ÄKTA Prime plus system and HiLoad 16/60 Superdex 75 gel filtration column were acquired from GE Healthcare for protein purification. Amicon ultra protein concentrators were purchased from Millipore (Bedford, Massachusetts, USA). All other chemicals were of analytical grade and purchased from commercial sources.

### Purification and crystallization of AVCP

Cloning, expression, purification, and crystallization of AVCP have been described previously [29]. Briefly, the gene encoding the carboxyl terminal domain of AVCP (residues 110–267) was amplified and cloned into the pET28c expression vector. The protein was over-expressed in *E. coli* Rosetta (DE3) by induction with isopropyl β-D-1-thiogalactopyranoside (IPTG). The protein was purified using Ni-NTA beads followed by TEV protease treatment to remove the His-tag. The His-tag cleaved protein was further purified by reverse Ni-NTA column and HiLoad 16/60 prep grade Superdex 75 size exclusion chromatography. The purified protein was concentrated up to ∼10 mg/ml for crystallization. Crystals were obtained in 28% w/v polyethylene glycol monomethyl ether 2000 containing 100 mM Bis-Tris (pH 6.5) at 20°C. Further, the native protein crystals were soaked in mother liquor containing 10 mM dioxane to obtain the AVCP-dioxane complex.

### Data collection

The crystals were cryo-protected by soaking in mother liquor containing 15% (v/v) glycerol and 12.5% (v/v) ethylene glycol. High resolution data were collected using Cu Kα radiation generated by a rotating-anode generator (Bruker-Nonius Microstar H) equipped with MAR345 imaging plate detector. The diffraction data were indexed, integrated and scaled using the HKL-2000 package [30].

### Structure determination and refinement

The structure was solved by the molecular replacement method using the Molrep program of the CCP4 program suite [31], [32]. The crystal structure of Sindbis virus capsid protein (SCP) (PDB ID: 1KXA) [33] was used as a search model for structure solution. Further refinement was carried out by the REFMAC program [34] of the CCP4 suite and COOT [35]. Six groups of TLS were chosen according to TLSMD server and used for anisotropic refinement of the molecule [36]. Iterative cycles of refinement were performed and interspersed with visual inspection and manual adjustments to obtain acceptable values of R_cryst_ and R_free_. Geometric restraints for the dioxane molecule were generated using ProDRG, an automated topology generation server [37]. The generated dioxane molecule was closely fitted into 2*F_o_ – F_c_* electron density and then included in following fitting and refinement cycles. PROCHECK was used for the evaluation of stereo-chemical properties of the model [38]. Visualization of the refined model and model building were carried out in COOT and the figures were prepared using PyMol [39].

### Molecular modeling

Molecular modeling of E1 and E2 was carried out by comparative methods and energy minimization using the program Swiss-Model in automated mode and MODELLER 9v8 [40], [41]. Following the five sequential steps (template selection from RCSB PDB database, sequence alignment, model generation, refinement and validation), three dimensional homology models of E1 and E2 of Aura virus were generated. The template was chosen on the basis of sequence identity and structural completeness, thus the complete VEEV structure (PDB ID: 3J0C) was used as a template for E1 and E2 model generation. MULTALIN server was used for the query sequence alignment to the template sequence [42]. After some manual corrections in the sequence alignment, five models were generated separately for both proteins using MODELLER. The quality of the model was assessed by PROCHECK. The best model was selected and energy minimization was performed using Swiss-PdbViewer 4.01 (http://spdbv.vital-it.ch/). Model validation was performed using ProSA energy plot and VERIFY-3D of the SAVES server [43], [44]. Finally, the crystal structure of AVCP and homology models of E1 and E2 were fitted into the cryo-EM density map of VEEV (EMDB ID: 5275) using fit-in-map module of Chimera software [45]. The figures of aligned sequences were generated using ClustalW and ESPript [46], [47].

### Accession numbers

The crystal structures of AVCP and its complex with dioxane have been deposited in Protein Data Bank with accession code 4AGK and 4AGJ respectively.

## Results and Discussion

The amino-terminal region of alphavirus CP is involved in the interaction with genomic RNA, while the C-terminal region acts as a serine protease having a chymotrypsin-like fold. Earlier reports suggested the amino-terminal region of this protein to be highly disordered based on the absence of its electron density in the crystal structure of full length CPs (Sindbis and Semliki Forest Virus CP) [48], [49]. However, mass spectroscopic analysis of the Semliki Forest virus CP crystals revealed that the molecular weight of protein molecules in the crystal is the same as that of the C-terminal protease domain [49]. Together, this provides evidence that the amino-terminal domain of Semliki Forest Virus CP is highly unstable and gets degraded during the process of purification or crystallization. Considering these facts, we expressed, purified and crystallized the C-terminal domain (residues 110–267) of AVCP for our studies.

The crystal structure of AVCP was determined using molecular replacement as described above. The structure of AVCP was refined to 1.81 Å resolution with R_cryst_ value of 17.6% and R_free_ value of 23.1%. Similarly, the structure of AVCP complexed with dioxane was refined to 1.98 Å resolution with R_cryst_ value of 17.9% and R_free_ value of 26.7% (Table 1). Both crystals belong to the space group *C2* with cell dimensions *a* = 79.6 Å, *b* = 35.2 Å, *c* = 49.5 Å having a Matthews coefficient of 2.0 Å^3^ Da^−1^ and the solvent content of 38.5%. 152 out of 158 residues have well defined electron density while electron density was missing at the N-terminus for the first six residues (110–115) in AVCP.

**Table 1:** Data collection and refinement statistics for AVCP in apo form and dioxane bound form.

	AVCP-apo	AVCP-dioxane bound
Crystallographic Data		
Space group	*C2*	*C2*
Cell dimensions (Å)	*a* = 79.6,*b* = 35.2, *c* = 49.5	*a* = 79.6,*b* = 35.2, *c* = 49.5
Resolution range (Å) (Last Shell)	50.0−1.81 (1.85−1.81)	50.0−1.98 (2.02−1.98)
Completeness (%) (Last Shell)	90.2 (70.7)	86.2 (45.7)
*R*merge† (%)(Last Shell)	5.7 (25.5)	4.8 (28.2)
Mean *I/σ*(*I*) (Last Shell)	22.6 (2.9)	20.3 (1.8)
No. of observed reflections	45053	24420
No. of unique reflections (Last Shell)	11410 (436)	7989 (160)
Molecules per asymmetric unit	1	1
Matthews coefficient (Å^3^ Da^−1^)	2.0	1.99
Solvent content (%)	38.5	38.5
Multiplicity (Last Shell)	3.9 (2.1)	3.1 (1.9)
Refinement		
No. of Residues	152	152
Water molecule	127	102
*R*cryst (%)	17.6	17.9
*R*free (%)	23.15	26.75
Average *B*-factor (Å^2^)	23.54	31.43
r.m.s.d on bond lengths (Å)	0.012	0.015
r.m.s.d on bond angles (Å)	1.567	1.926
Ramachandran plot (%)		
Preferred	98.0	95.33
Allowed	2.0	4.0
Outliers	0.0	0.67

† *R*merge = Σ*_hkl_* Σ*_i_* |*I*_i_(*hkl*)−[*I*(*hkl*)]|/Σ*_hkl_* Σ_i_ *I*_i_(*hkl*), where *I*_i_(*hkl*) is the *i*th observation of reflection *hkl* and [*I*(*hkl*)] is the weighted average intensity for all observations *i* of reflection *hkl*.

### Overall structure of AVCP

**Figure 1:**
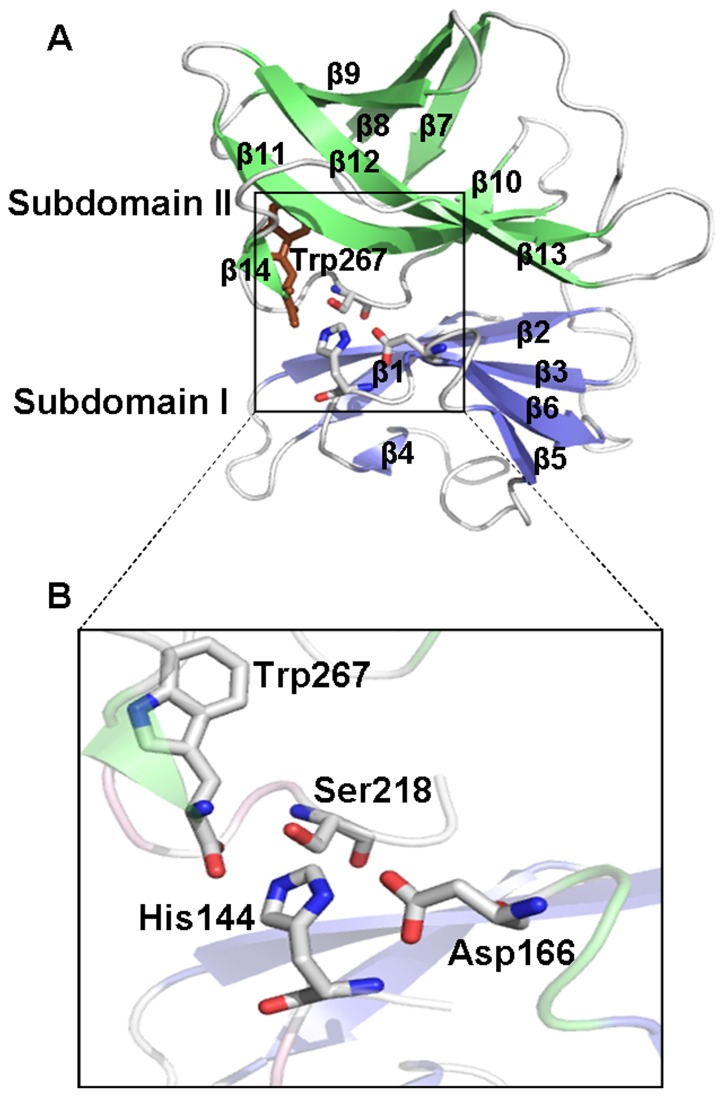
Structure of Aura Virus Capsid Protease. (**A**) Overall structure of the capsid protein with two β-barrels of subdomains I and II colored in blue and green respectively. The catalytic triad residues and Trp267 are shown in sticks; (**B**) Close-up view of the active site shows catalytic triad composed of Ser218, Asp166 and His144 along with the carboxy-terminal Trp267 approaching the active site.

**Figure 2:**
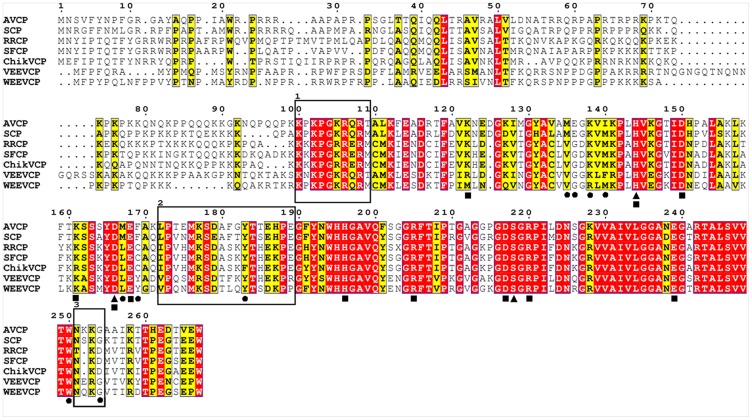
Multiple sequence alignment of AVCP with CPs from other alphaviruses. The conserved residues are shown in red background. The circles under the amino acids indicate the hydrophobic pocket residues interacting with the dioxane molecule while the triangles denote the catalytic triad residues. The residues forming salt bridges are denoted by squares below the residues. The motif responsible for specificity in encapsidation is highlighted in rectangular box 1. Interdomain flexible loop residues are shown in box 2 whereas the flexible loop separating the two pockets for E1 and E2 binding is shown in box 3. Capsid protein sequences used for alignment are: Aura virus (AVCP), Sindbis (SCP), Ross River (RRCP), Semliki Forest (SFCP), Chikungunya (ChikVCP), Venezuelan equine encephalitis (VEEVCP) and Western equine encephalitis (WEEVCP).

The overall fold of AVCP (C-terminal domain) consists of two β-barrel sub-domains similar to crystal structures of other alphavirus CPs [48], [49]. The sub-domains I and II consist of six and eight β-strands respectively, joined via a long linker loop region comprising of eighteen residues (Leu172-Glu189) (Figure 1). The twisted anti-parallel β-strands of individual sub-domains [β1 (118–122), β2 (128–135), β3 (138–142), β4 (148–149), β5 (160–162), β6 (167–171) of sub-domain I and β7 (190–194), β8 (197–202), β9 (205–209), β10 (221–223), β11 (229–238), β12 (242–250), β13 (256–259) and β14 (265–266) of sub-domain II] form the Greek key motif that is a characteristic of chymotrypsin-like serine proteases. Six salt bridges confined to individual sub-domains are reported which might be responsible for compact structure and stability. Three salt-bridges (Asp150-Lys122, Glu168-Lys161 and Asp166-His144) are confined to sub-domain I whereas other three Asp217-Arg220, Asp217-His196 and Glu239-Arg205 are confined to sub-domain II (Figure 2).

Alphavirus CP possesses *cis*-autoproteolytic activity for its cleavage from the rest of the structural polyprotein during the viral infection process [50]. Once cleaved, the mature CP does not cleave other CPs further *in trans* due to the binding of the C-terminal Trp267 residue at the P1 position near the active site [48], [51]. Due to conserved structural characteristics, the active site architecture in all members of the serine protease family appears to be quite similar. As a conserved feature of all the members of serine protease family, AVCP contains a catalytic triad formed of His144, Ser218 and Asp166 residues. The triad is located in the cleft between the β-barrel sub-domains (Figure 1). The backbone amides of Ser218 (the active site residue) and Gly216 form the oxyanion hole and act as donor of backbone hydrogen bond.

AVCP appears to be monomeric both in the crystal structure as well as in solution as observed by size-exclusion chromatography [29]. In the crystal structures of Sindbis capsid protein (SCP) and Semliki Forest capsid protein (SFCP), a crystallographic dimer with similar dimeric interface has been reported [48], [49]. Interestingly, fit of SFCP crystal structure in the cryo-EM density suggests that molecular contacts similar to the dimer interface interactions are present in the virions. However, AVCP does not show dimerization property even though the residues involved in crystallographic dimer formation in SCP are conserved in the sequence of AVCP (Figure 2, 188-Pro-Glu-Gly-Phe-Tyr-Asn-193 Gly197, Ala198). In addition to this, a single mutation at conserved Phe188Gly in SCP resulted in the disruption of the crystallographic dimer but had no effect on virus replication [33], [51]. Thus, the biological functions of the conserved dimer interface residues in the virus core structure formation need to be confirmed by additional mutational and structural studies.

### Comparative analysis with capsid proteases from different alphaviruses

In order to attain a detailed comparative analysis, the superposition of AVCP with CPs from other alphaviruses was performed. The homology search for AVCP using blastp tool of National Center for Biotechnology Information (NCBI) (http://blast.ncbi.nlm.nih.gov/Blast.cgi) against the PDB database showed the hits of crystal structure of alphavirus capsid proteins with range 58% to 79% sequence identity. However, when the 3D coordinates of AVCP were used to search structural homologs using DALI server [52], the search results showed the most structurally conserved proteins of serine protease family including flavivirus NS3 protease, elastase, α-lytic protease and chymotrypsin. Structural similarity between AVCP and flavivirus NS3 protease indicates that these may have evolved from a common chymotrypsin-like serine protease. Similarly, divergent evolution of alphaviruses and flaviviruses from a common ancestor has been suggested based on the structural homology between fusion glycoproteins E1 and E from alphaviruses and flaviviruses respectively [53], [54].

**Figure 3:**
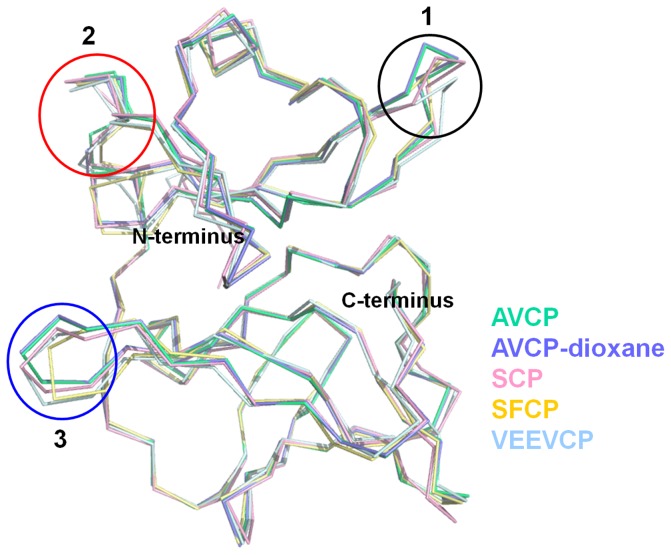
Structural alignment of CPs from different alphaviruses. Structure alignment of both apo (green) and complex (blue) form of AVCP with SCP (pink), SFCP(yellow) and VEEVCP (cyan) with PDB IDs: 1KXA, 1VCP and 1EP5 respectively showing structural variation. Three circles show loop regions of high variability that include inter-domain flexible loop region (Circle 2) and flexible loop that separate the two pockets responsible for binding to E1 and E2 (Circle 3).

The β-barrel is well conserved among serine protease family; however, the size of the β-sheets varies. Superimposition of the apo form of AVCP with CP structures from other alphaviruses [SCP (RMSD = 0.481 Å), SFCP (RMSD = 0.468 Å) and VEEVCP (RMSD = 0.540 Å)] illustrates a high degree of structural similarity. Most notable variations are seen within the three loop regions (Figure 3). In SFV, fitting of CP crystal structure into EM density suggests that these loop regions (SFV residues 125–128, 171–181, 252–255) are involved in CP-CP contacts in virions [49]. Particularly, AVCP does not possess any helix in the structure, whereas CPs of other alphaviruses possess a 3_10_ and/or an α-helix. The inter-domain loop region connecting the beta-sheets is highly flexible and adopts different conformations (Circle 2, Figure 3). Sequence comparison shows that this inter-domain loop shares relatively less conserved region with respect to overall sequence identity (Box 2, Figure 2). The C-terminus of AVCP terminates with a short β-strand consisting of Val265-Glu266 residues that has been proposed to keep the C-terminal Trp residue in a static position near the active site [51]. This C-terminus Trp occupies the same spatial position in all alphaviruses and resides near the active site needed for autoproteolysis.

Apart from structural comparison, sequence alignment also provides interesting information regarding encapsidation of alphaviruses. The N-terminal motif Lys97-Met106 of SCP (corresponding to Lys100-Thr109 of AVCP) was found to be responsible for specific encapsidation as this motif has been shown to be required for specificity of genomic RNA encapsidation [7]. However, even though the motif is highly conserved in all alphaviruses, Aura virus encapsidates both genomic RNA and subgenomic RNA in virus particles [55] (Figure 2). Therefore, the possible explanations for encapsidation of the subgenomic RNAs by Aura virus could be that the subgenomic RNAs of Aura virus may contain a packaging signal. The identification of a packaging signal, if any, in Aura virus subgenomic RNA needs to be investigated further.

### Interaction analysis of E1 and E2 glycoproteins with capsid protease

**Figure 4:**
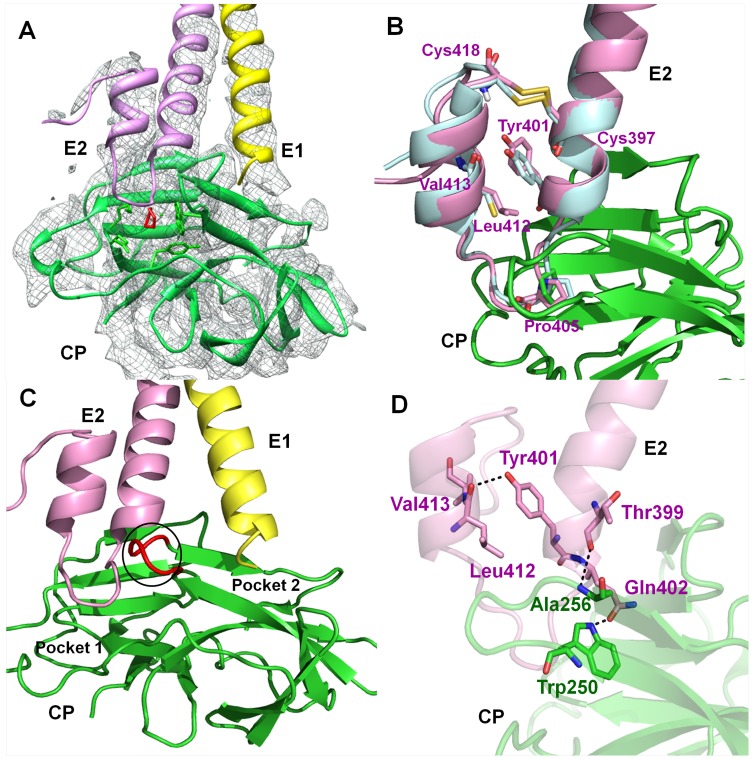
CP-glycoproteins interaction along with analysis of different amino acids in cdE2. (**A**) Representation of the crystal structure of AVCP-dioxane complex and the homology models of E1 and E2 glycoproteins fitted into the cryo-EM electron density map of VEEV (EMDB ID: 5275). AVCP, E1 and E2 are shown in green, yellow and pink respectively; bound dioxane is shown in red while the cryo-EM density map is denoted by gray; (**B**) Structural alignment of cdE2 predicted structure from Aura virus (pink) and VEEV (blue) showing Tyr401 residue oriented towards other helix away from CP and the disulfide bond (yellow) within the helix-loop-helix motif of E2. The Pro405 interaction with the hydrophobic pocket present on the surface of the AVCP crystal structure is also shown; (**C**) Cartoon view of the AVCP-glycoproteins interaction. Binding of Aura virus E2 and E1 cytoplasmic tails to different pockets (P1 and P2 respectively) found in close proximity at the surface of AVCP. E2 shows the helix-loop-helix structural architecture and E1 has a helical structure that interacts with AVCP. The loop separating both interacting pockets is shown in red color; (**D**) The critical polar interactions between E2 and capsid as well as within the helix-loop-helix motif of cdE2 are shown in dotted lines. E2 residues are shown in pink while the AVCP residues are displayed in green. The interacting residues are shown as sticks.

The recent cryo-EM structure of VEEV opened up the possibility to explore the interaction patterns of glycoproteins with CP [27]. The VEEV structure revealed that the cytoplasmic tail of E2 interacts with the hydrophobic pocket of capsid. Furthermore, Tang et al. (2011) elucidated the interaction at molecular level by the cryo-EM structure of Sindbis virus and identified by mutagenesis that Arg393 and Glu395 residues of cdE2 and Tyr162 and Lys252 of capsid as the key residues for interaction [26]. However, the exact interaction is not yet clear. To throw light on the mode of interaction, we fitted the crystal structure of AVCP and homology models of the cytoplasmic tails of E1 and E2 trans-membrane glycoproteins into the cryo-EM density map of VEEV (Figure 4A). The superposition of Aura virus E1 and E2 cytoplasmic tails to that of VEEV shows RMSD of 0.206 Å and 0.578 Å respectively (Figure 4B).

**Figure 5:**
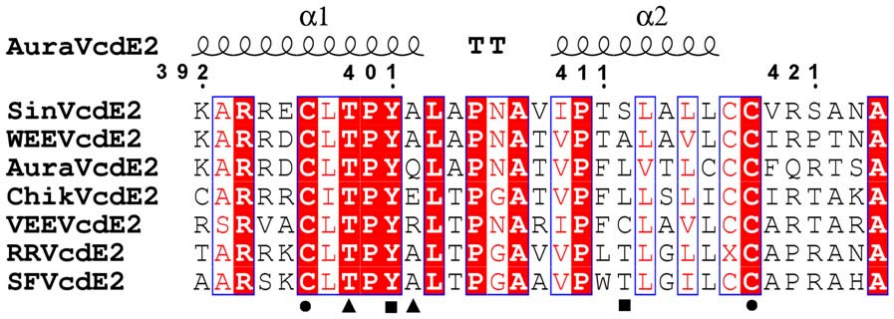
Multiple sequence alignment of the cytoplasmic domain of E2 (cdE2) from different alphaviruses. The secondary structure of 33 residues from Aura virus cdE2 displays a helix-loop-helix structure. Highly conserved residues are in red background. The circles under residues denote the disulfide bridge forming Cys residues, squares indicate the residues responsible for polar interactions within the E2 helix-loop-helix motif and the triangles represent the residues of E2 making polar interactions with CP. cdE2 sequences used for alignment are: Sindbis (SinVcdE2), Western equine encephalitis (WEEVcdE2), Aura (AuraVcdE2), Chikungunya (ChikVcdE2), Venezuelan equine encephalitis (VEEVcdE2), Ross River (RRVcdE2) and Semliki Forest (SFVcdE2).

The C-terminus of E2 is highly conserved in all alphaviruses (Figure 5) and possesses a tightly folded helix-loop-helix motif that makes a globular structure to fit into the hydrophobic pocket of AVCP (Figure 4). The conserved residue Tyr400 in Sindbis virus was proposed to be responsible for E2-capsid interaction [21], [56]. Crystal structure of SCP showed the binding of N-terminal arm hydrophobic residues Leu108 and Leu110 in the hydrophobic pocket of neighboring CP molecule [48]. Additionally, mutational analysis of Sindbis virus showed that for successful virus propagation only hydrophobic residues could substitute the conserved Tyr400 (Tyr401 in Aura virus) [21]. Based on these observations, it was assumed that Tyr400, a conserved residue of cdE2 binds into the hydrophobic pocket of CP. However, in the generated model of Aura E2, Tyr401 makes a hydrogen bond with Leu412 and forms hydrophobic contacts with Leu412 and Val413 of the opposite α-helix. Hence, Tyr401 residue of cdE2 is oriented away from the hydrophobic pocket of capsid towards the other helix in helix-loop-helix structure (Figure 4D). Thus, it depicts that Tyr401 may play a critical role in maintaining the helix-loop-helix structure of cdE2. Additionally, Jose et al. (2012) recently demonstrated that Cys residues in cdE2 are critical for the budding process of alphaviruses [25], as these have been proposed to undergo palmitoylation to develop a hydrophobic region that facilitates interaction with capsid [57]. Structural elucidation reveals that four Cys residues i.e. Cys397, Cys416, Cys417 and Cys418 are present in the C-terminal tail of Aura virus E2. Interestingly, Cys397 is found to make a disulfide bridge with Cys418 to establish a stable C-terminal lobe structure (Figure 4B). Moreover, we have also analyzed the cryo-EM structure of VEEV and found that the cytoplasmic tail of VEEV E2 also contains a disulfide bond at the same position. This disulfide bridge is expected to be formed in cdE2 of all alphaviruses as both the disulfide forming cysteine residues are conserved at the corresponding positions (Figure 5). Furthermore, the proposed structural role of cdE2 disulfide bridge is supported by mutational data of Sindbis virus cdE2 Cys417 mutation (Cys418 in Aura virus) which shows that Cys417Ala mutation slows down virus replication and decreases the infectious virus yield [58].

Structural analysis of the interactions between cdE2 and capsid reveals that the loop region of the helix-loop-helix motif of cdE2 docks into the hydrophobic pocket of AVCP (Figure 4A) and the pyrrolidine ring of Pro405 of cdE2 makes cryo-EM structure of VEEV shows that the conserved proline of cdE2 occupies the same position as Pro405 of Aura virus cdE2 in the hydrophobic pocket of CP (Figure 4B). These structural investigations suggest that Pro405 might play a crucial role in alphavirus budding process. Our observation is supported by mutational studies in Sindbis virus cdE2 where Pro404Ala substitution (corresponding to Pro405 of Aura virus cdE2) results in slow-growth phenotype. Also, this mutant was found to be more thermolabile than the parental wild type virus [57], [58]. In addition, few polar contacts are found to be involved in stabilizing the E2-capsid interaction. Thr399 and Gln402 of E2 glycoprotein show polar contacts with Ala256 and Trp250 of AVCP respectively (Figure 4D). Furthermore, the cryo-EM structure also revealed the mode of E1-capsid interaction. E1 glycoprotein in Aura virus is expected to interact in a small depression in the capsid protein (P2 pocket), which is separated from the E2-interacting hydrophobic pocket (P1 pocket) by a small flexible loop stretching from Asn251 to Ala255 of AVCP (Figure 3, Figure 4C). Although the exact mode of interaction is not clear, it can be assumed that the E1 cytoplasmic tail extends towards the capsid with limited polar interactions with the E2 cytoplasmic tail.

### Inhibition strategy to disrupt capsid and glycoprotein interaction

The apo form of AVCP crystal structure reveals the presence of hydrophobic pocket composed of conserved residues on the surface of CP, which has also been reported in both the crystal structures of SCP and SFCP. Interestingly, a solvent-derived dioxane molecule was found to be bound to this hydrophobic pocket in the crystal structure of SCP [22], suggesting that dioxane derivatives may prevent capsid–E2 hydrophobic interactions with the capsid hydrophobic pocket (Figure 4B). Likewise, analysis of the binding. As expected, dioxane-derived molecules showed antiviral properties against Sindbis virus [23], [24]. AVCP is the third crystal structure of alphavirus capsid protein, with SCP and SFCP, that confirms the presence of a structurally conserved hydrophobic pocket on the CP surface. Therefore, dioxane and its derivative molecules are expected to bind the hydrophobic pocket and inhibit virus budding by disrupting capsid–E2 interaction in all alphaviruses including Aura virus. Therefore, we determined the structure of AVCP in complex with dioxane. The superposition of AVCP-apo and AVCP-dioxane structures showed RMSD of 0.133 Å (Figure 3) and dioxane binding does not affect the overall structure of AVCP.

**Figure 6:**
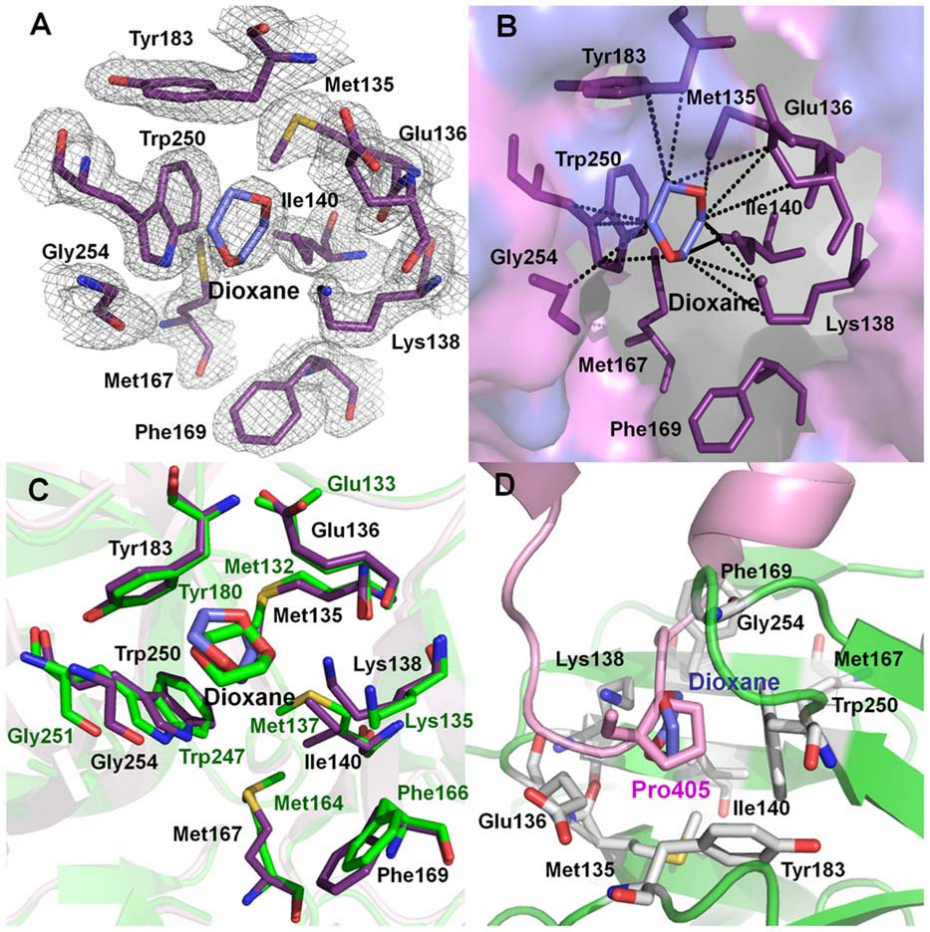
AVCP-dioxane complex structure showing the dioxane binding pocket and the position of Pro405 from cdE2. (**A**) Hydrophobic pocket showing a bound dioxane molecule on the surface of AVCP crystal structure with 2*F*_obs_-*F*_calc_ electron density contoured at 1σ level. The residues present in the hydrophobic pocket are shown in violet and the dioxane molecule in blue. The electron density of the pocket is shown in gray; (**B**) The hydrophobic pocket of the AVCP complexed with dioxane as revealed by crystal structural studies. The interacting residues (violet) and dioxane molecule (blue) are shown as sticks. The hydrophobic interactions shown in dashed lines were analyzed by ligand explorer of RCSB PDB (ligpro.sdsc.edu); (**C**) Superimposition of dioxane bound forms of AVCP (violet) and SCP (1WYK) (green) crystal structures highlighting the hydrophobic pocket residues; (**D**) The dioxane molecule (blue) in the crystal structure of AVCP-dioxane complex occupies the same position as Pro405 (pink) present in the helix-loop-helix motif of cdE2. A homology model of cdE2 was combined with the AVCP crystal structure. The residues in AVCP that are involved in the interaction are shown as gray sticks.

The electron density for the dioxane molecule is clearly visible in the difference Fourier map in the hydrophobic pocket of AVCP (Figure 6A). The dioxane complex structure described in this report is the second structure of dioxane-CP complex among alphaviruses after SCP (PDB ID: 1WYK) [22]. Dioxane binds to the hydrophobic pocket in the AVCP-dioxane complex in almost the same orientation as that of the SCP-dioxane structure with similar hydrophobic interactions. In the dioxane binding hydrophobic pocket, AVCP and SCP share highly conserved residues. Eight residues out of nine in this hydrophobic pocket are strictly conserved whereas Met137 of SCP is substituted with Ile140 in AVCP (Figure 6C). In SCP, four residues (i.e. Met132, Glu133, Tyr180 and Trp247) contribute to major hydrophobic interaction with dioxane. However, in AVCP, seven residues (Met135, Glu136, Lys138, Ile140, Tyr183, Trp250 and Gly254) are in hydrophobic contact with the bound dioxane molecule (Figure 6B). Though SCP possesses the same number of conserved residues (except Met137), Lys135 and Gly251 are positioned slightly away from dioxane, rendering poor hydrophobic interactions with it (Figure 6C). Thus, binding of dioxane to the CP hydrophobic pocket can be considered as characteristic for all the alphaviruses.

Further interaction analysis of cdE2 and capsid reveals that dioxane occupies the same position as would be occupied by the pyrrolidine ring of Pro405 of cdE2 (Figure 6D). Thus, competitive inhibition could be a plausible mechanism by which dioxane and its derivatives may disrupt capsid-E2 interaction in alphaviruses including Aura virus by obstructing the hydrophobic interaction of conserved Pro405 with CP. This assumption is based on the dioxane bound AVCP structural data where dioxane occupies the hydrophobic pocket on CP and structurally mimics the hydrophobic pyrrolidine ring of Pro405 in loop region of E2. Additionally, structural evidence shows binding of a dioxane molecule to the equivalent hydrophobic pocket on SCP and inhibition of Sindbis virus replication by dioxane-based inhibitors supports this [22]–[24].

Finally, our structural analysis revealed the key residues involved in the stabilization of the helix-loop-helix structure of cdE2 that were previously identified to be important for CP-glycoprotein interaction. In addition, we proposed Pro405 of E2 to be directly interacting with the capsid hydrophobic pocket. The crystal structure of AVCP in complex with dioxane showed a dioxane molecule bound to the hydrophobic pocket of CP. The conservation of alphavirus capsid hydrophobic pocket and binding of dioxane to the same hydrophobic pocket in the crystal structure of CP from two alphaviruses (SCP and AVCP) signifies that a broad-spectrum inhibitor that targets CP-E2 interactions during virus budding can be developed. In addition, based on Pro405 interaction with CP, a structure-based approach can be employed for the design and development of novel pyrrolidine derived alphavirus-specific antivirals.

### Conclusions

The interaction of glycoproteins with the capsid protein is crucial for alphavirus budding process. Recent cryo-EM studies revealed the overall interaction pattern of these proteins. In the present study, we have determined the crystal structure of AVCP and generated homology models of E1 and E2 from Aura virus. To investigate CP-glycoprotein interactions in more details, the AVCP crystal structure and homology models of E1 and E2 were fitted into the cryo-EM density map of VEEV. The structural elucidation and extensive interaction analysis revealed several interesting features. The cytoplasmic tail of E2 is predicted to contain a helix-loop-helix topology that may play an important role in CP-E2 interaction. Previous studies have suggested that residues Tyr401, Cys397 and Cys418 in E2 are important for interaction of E2 with CP. Interestingly, comparative modeling of Aura virus E2 suggests that these residues play a major role in the stabilization of the helix-loop-helix topology of cdE2. Additionally, comparative modeling and fitting studies suggest that Pro405, a conserved residue present in the loop region of the helix-loop-helix motif of Aura virus interacts directly with the hydrophobic pocket of CP. The crystal structure of the AVCP-dioxane complex has also been determined and the most striking feature observed in it is that dioxane exclusively occupies the position of Pro405 in the CP hydrophobic pocket. Thus, it is proposed that dioxane based-derivative molecules would compete with binding of Pro405 in the CP hydrophobic pocket and disrupt the CP-cdE2 interaction in Aura virus. The proposed role of various Aura virus cdE2 residues needs to be confirmed by mutational and crystal structural studies of capsid-cdE2 peptide complexes. The structure of AVCP that elucidates the hydrophobic pocket, comparative modeling of glycoproteins and cryo-EM fitting studies that depict the binding pattern of CP-glycoproteins will define a valuable roadmap for developing a new strategy for antiviral drug discovery.
